# Fluorescent and Bioluminescent Reporter Myxoviruses

**DOI:** 10.3390/v8080214

**Published:** 2016-08-04

**Authors:** Christina A. Rostad, Michael C. Currier, Martin L. Moore

**Affiliations:** 1 Department of Pediatrics, Emory University School of Medicine, Atlanta, GA 30307, USA; Christina.rostad@emory.edu (C.A.R.); mgcurri@emory.edu (M.C.C.); 2 Children’s Healthcare of Atlanta, 1405 Clifton Road, Atlanta, GA 30322, USA

**Keywords:** fluorescent reporter virus, bioluminescent reporter virus, paramyxovirus, orthomyxovirus

## Abstract

The advent of virus reverse genetics has enabled the incorporation of genetically encoded reporter proteins into replication-competent viruses. These reporters include fluorescent proteins which have intrinsic chromophores that absorb light and re-emit it at lower wavelengths, and bioluminescent proteins which are luciferase enzymes that react with substrates to produce visible light. The incorporation of these reporters into replication-competent viruses has revolutionized our understanding of molecular virology and aspects of viral tropism and transmission. Reporter viruses have also enabled the development of high-throughput assays to screen antiviral compounds and antibodies and to perform neutralization assays. However, there remain technical challenges with the design of replication-competent reporter viruses, and each reporter has unique advantages and disadvantages for specific applications. This review describes currently available reporters, design strategies for incorporating reporters into replication-competent paramyxoviruses and orthomyxoviruses, and the variety of applications for which these tools can be utilized both in vitro and in vivo.

## 1. Introduction

Fluorescent proteins (FPs) were first described in the 1960s when the green fluorescent protein (GFP) was isolated from the *Aequorea victoria* jellyfish [1]. GFP was first sequenced and cloned in 1992 [2], and it was first used as a reporter protein to mark gene expression in *Escherichia coli* and in *Caenorhabditis elegans* in 1994 [3]. Since that time, an array of naturally occurring fluorescent proteins and genetically engineered derivatives have been developed that encompass nearly the entire the spectrum of visible wavelengths. As reporters, these proteins have made the monitoring and visualization of numerous intracellular events with high temporal and spatial resolution possible, ranging from gene expression, to protein localization and interaction, to cellular signaling and trafficking. Using virus reverse genetics, fluorescent proteins have also been incorporated into an increasing number of replication-competent viruses, elucidating aspects of viral pathogenesis, tropism, and transmission. Bioluminescent proteins have similarly been incorporated into live viruses and have recently gained traction as tools for characterizing viral infections, especially in vivo [4]. The purpose of this review is to examine the properties of fluorescent and bioluminescent proteins, their applications in replication-competent paramyxoviruses and orthomyxoviruses, and considerations for their design and development.

## 2. Characteristics of Fluorescent and Bioluminescent Proteins

**Figure 1:**
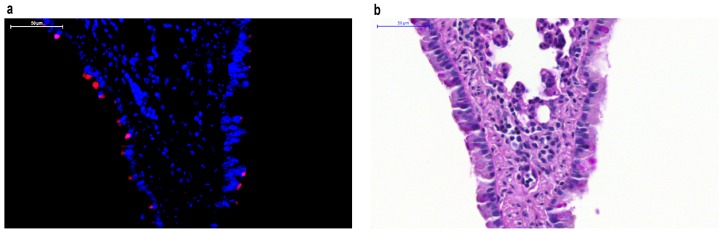
Respiratory syncytial virus (RSV) labeled with monomeric red fluorescent protein mKate2 in mouse lower respiratory tract epithelial cells. (**a**) RSV localizes to the apical surface of respiratory epithelial cells and can be tracked in vivo in paraffin-embedded tissues using mKate2 as demonstrated with fluorescent imaging using 4”,6-diamidino-2-phenylindole (DAPI) counterstaining; (**b**) Periodic Acid-Schiff (PAS) stain of the same lung specimen.

Fluorescent proteins of the GFP family are homologous genetically-encoded proteins with intrinsic fluorescence which is not dependent on exogenous substrates other than molecular oxygen [5]. The structure of GFP consists of 11 β-sheets which form a barrel around a centrally located chromophore [6]. The chromophore is derived from three amino acids within the polypeptide sequence, which undergo unique post-translational modifications at residues 65–67 (Ser-Tyr-Gly in *Aequorea victoria* GFP). The central location of the chromophore renders it relatively stable to changes in temperature, pH, and physical stress. Naturally occurring fluorescent proteins of the GFP family have been described which encompass a broad palette of colors, including red (DsRed) [7], yellow (zFP538) [7], and green-to-red (the photoconvertible protein Kaede) [8]. Mutagenesis of residues within naturally occurring chromophores has enabled the development of unique fluorescent proteins with broadened spectral diversity. An important example has been the development of the monomeric, far-red fluorescent proteins Katushka (mKate) [9] and its brighter variant mKate2 [10] via random and site-directed mutagenesis of eqFP586 from the sea anemone *Entacmaea quadricolor*, which have been used in tissue (Figure 1) and whole body imaging. The genetically engineered fluorescent proteins also have a range of brightness, maturation rate, photostability, pH stability, and tendency for oligomerization; each of these properties may play an important role in the selection of an appropriate protein for a specific application [11]. 

Bioluminescent proteins (BPs) are luciferase enzymes, which react with substrates to produce visible light. They are distinct from fluorescent proteins, which have intrinsic chromophores that absorb light and re-emit it at lower wavelengths. Although bioluminescent and fluorescent proteins are utilized in many analogous applications, BPs have established a particular niche in the live bioluminescent imaging of small animal models [12]. BPs are advantageous in live imaging because they are highly sensitive (can detect down to 10 [2] virus plaque forming units/mL) [13] and have high signal-to-noise ratios. One disadvantage of BPs is their requirement for an exogenous substrate, which must be delivered to cells or tissues at sufficient concentration to generate signal. The firefly luciferase has been the most commonly utilized bioluminescent protein for in vivo imaging because its substrate luciferin has a good pharmacokinetic profile, sufficient bioavailability, and a red-shifted emission spectrum [14]. Recently, a novel small luciferase enzyme called NanoLuc and its substrate furimazine were engineered from the deep-sea shrimp *Oplophorus gracilirostris* [15]. Purified NanoLuc produced 150-fold greater luminescence than either firefly or *Renilla* luciferases and exhibited prolonged enzyme stability and signal duration in vitro [15]. The utility of this promising new reporter has been demonstrated with in vivo imaging of viral infections in small animals, although the pharmacokinetic profile of the substrate furimazine has not yet been fully elucidated and the blue-shifted emission wavelength may be disadvantageous [16,17]. 

## 3. Reverse Genetics Systems for Paramyxoviruses and Orthomyxoviruses

The advent of virus reverse genetics has enabled the incorporation of genetically encoded reporter proteins into replication-competent viruses. The first negative-sense RNA virus to be rescued entirely from cloned cDNA was rabies virus in 1994 [18]. By 1996, vesicular stomatitis virus [19] and three paramyxoviruses, measles [20], Sendai virus (murine parainfluenza 1) [21], and respiratory syncytial virus [22] had been rescued by similar methods. Reverse genetics systems for all genera of the *Paramyxoviridae* family have now been successfully developed. 

*Paramyxoviridae* are non-segmented, single-stranded, negative-sense RNA viruses which consist of two subfamilies: *Paramyxovirinae* and *Pneumovirinae*. Clinically significant paramyxoviruses which are pathogenic in humans include measles (MeV), mumps, the highly virulent Nipah and Hendra viruses, and respiratory viruses such as human metapneumovirus (HMPV), human respiratory syncytial virus (RSV), and the human parainfluenza viruses. Paramyxoviruses consist of 6–10 genes, which are transcribed by the viral ribonucleoprotein (RNP) complex down a transcriptional gradient, with the 3’ genes transcribed in higher quantities than the 5’ genes. Unlike positive-sense viruses, negative-sense viral RNA is not infectious, as it requires a functional RNP complex to perform transcription and replication. Reverse genetics approaches have therefore relied upon co-transfection of genes encoding proteins of the RNP complex (nucleoprotein, phosphoprotein, and large polymerase) with viral cDNA under the control of a promoter, often T7 polymerase. The T7 polymerase is typically supplied either by a constitutively expressing cell line (e.g., BSR-T7/5 cells), a co-transfected plasmid, or co-infection with a T7 polymerase-producing virus. Alternative approaches have implemented inherent cellular RNA polymerase I or II under control of the CMV promoter to transcribe viral genes [23,24].

*Orthomyxoviridae* are segmented, single-stranded, negative-sense RNA viruses. The most important human pathogens in the *Orthomyxoviridae* family are the influenza viruses, which cause annual seasonal pandemics worldwide. Early attempts to rescue recombinant influenza virus via reverse genetics utilized helper viruses to supply viral transcription and replication machinery [25,26]; however, these early approaches required selection against the helper viruses, which was often associated with technical challenges. In 1999, Neumann et al. successfully generated influenza virus entirely from cloned cDNA by co-transfecting eight plasmids encoding the viral gene segments under the control of RNA polymerase I along with four plasmids encoding the components of viral RNP (N, PB1, PB2, and PA) under the control of RNA polymerase II [27]. In 2000, Hoffmann et al. then developed an eight-plasmid cDNA transfection system for the rescue of influenza A by inserting viral RNA flanked by RNA polymerase 1 promoter and terminator sequences between RNA polymerase 2 promoter and polyadenylation sites oriented in the reverse direction. This schema enabled the synthesis of both negative- and positive-sense RNA from the same cDNA template and reduced the number of plasmids needed for successful viral rescue to eight [28]. Because eukaryotic cell lines approved for vaccine production (e.g., Vero cells) have low transfection efficiencies, further efforts were made to decrease the number of plasmids required for influenza virus production. In 2005, Neumann et al. successfully rescued influenza virus from a single plasmid encoding all eight viral RNA segments under the control of RNA polymerase I. Virus yield was further increased when this plasmid was co-transfected with helper plasmids encoding the components of the RNP complex [29].

## 4. Design of Reporter Paramyxoviruses and Orthomyxoviruses

### 4.1. Selection of Fluorescent Proteins

There is not a universal approach to the design of fluorescent and bioluminescent reporter viruses using reverse genetics. Key design considerations include the selection of the appropriate reporter proteins and expression strategies for specific applications.

### 4.2. Brightness

The brightness of a chromophore is proportional to the product of the extinction coefficient (ε)—which is the capacity for light absorption at a specific wavelength—and the quantum yield (QY), which is the number of fluorescent photons emitted per photon absorbed. A bright fluorophore can be advantageous in that it can increase the signal-to-noise ratio and reduce the amount of excitation light needed, thereby decreasing phototoxic effects. The brightest fluorophores tend to have yellow-green emission spectra, although many naturally occurring fluorophores have been genetically modified to increase brightness. One commonly used example is the enhanced green fluorescent protein (eGFP), which contains a single S65T mutation and generates a five-fold brighter fluorophore with a faster maturation rate than native GFP [30]. 

For in vivo imaging, fluorophores with red and far-red shifted excitation and emission spectra are preferable because whole tissue absorbs light up to 600 nm in wavelength. Investigators have therefore utilized site-directed mutagenesis and directed evolution approaches to generate brighter red and far-red fluorescent proteins such as mKate2 for utilization in whole animal imaging [9,31,32].

### 4.3. Photostability

All fluorescent proteins eventually undergo irreversible photobleaching after prolonged or high-intensity illumination. Thus, photostability is of particular importance when selecting a fluorescent protein for experiments with long time series or multiple sequential images. Shaner and colleagues [33] developed an assay for screening libraries of fluorescent proteins for enhanced photostability. Using this method, they generated TagRFP-T and mOrange2, which were 9-fold and 25-fold more photostable than their parental molecules, respectively. A uniform methodology to quantify fluorophore photostability is lacking. Some experts therefore recommend direct comparison of fluorophore photostability for a particular application prior to embarking on a large-scale experiment [11].

### 4.4. Maturation Time

Once a fluorescent protein has been translated, it must undergo folding and multiple post-translational modifications in order to become fluorescent. This process is called maturation, and the time it requires is the maturation time. Most fluorescent proteins have maturation times of several minutes to 1–2 h. The maturation rate may become important in experiments with short time courses relative to the maturation time of the fluorophore. In contrast, fluorescent proteins with prolonged maturation times [34] or with maturation processes that progress through intermediate chromophores [35] known as “Timers” have been exploited to study the dynamics of intracellular processes on larger time scales [36].

### 4.5. pH Stability

The pH stability of a fluorescent protein is expressed as the pK_a_, which is the pH at which the FP fluorescence is half its maximal brightness. Because pH varies among intracellular compartments, fluorophore pH stability should be considered when labeling proteins that traverse these organelles. The pK_a_ of wild-type GFP is approximately 4.8, but GFP has a broad range of pH stability from 6 to 10 [37]. Interestingly, some GFP variants, which have been mutated to optimize spectral properties have increased acid sensitivity. This is true of eGFP, which has a pK_a_ of approximately 6 [30]. Fluorescent proteins with enhanced pH stability—such as mTagBFP2, with a pK_a_ of 2.7—have been developed [38]. However, the imaging of lysosomes and other acidic organelles is still fraught with challenges [39]. As with other properties, the pH sensitivity of fluorescent proteins has been exploited to develop intracellular pH sensors [40].

### 4.6. Oligomerization

The tendency of oligomerization is inherent in many fluorescent proteins, which can lead to protein aggregation and attenuation of signal. When these FPs are fused to viral proteins, oligomerization may also render the protein of interest non-functional. While this may be of lesser concern for in vitro assays such as high throughput screenings, it may substantially affect the monitoring of intracellular processes. Wild-type GFP has only a weak tendency to dimerize at high concentrations. In contrast, the first red fluorescent protein discovered was DsRed from *Discosoma* sp., which forms obligate tetramers. The derivation of a monomeric red fluorescent protein from DsRed was elusive until 2002 when Campbell et al. used a directed evolution approach to impede the interaction of each subunit interface to generate mRFP1 [41]. From mRFP1, several monomeric fluorescent proteins have since been generated known collectively as the “mFruits” with improved brightness and photostability and broader excitation and emission spectra compared to the first generation mRFP1 [42]. Monomeric versions of fluorescent proteins are now available across the spectrum of visible light. 

### 4.7. Selection of Luciferase Proteins

As mentioned above, bioluminescent reporter proteins generally have higher sensitivity and signal-to-noise ratio than fluorescent proteins, primarily due to the intrinsically low background bioluminescence observed in cells and tissues. This is useful for in vivo imaging applications, although the luciferase substrate must be administered and must achieve sufficient biodistribution to generate signal. Important determinants in selecting a bioluminescent label include the size of the luciferase, the intensity and wavelength of luminescence produced, and the bioavailability and ease of administration of the substrate. 

Many different luciferases have been identified in nature, but three have been studied extensively and implemented for biomedical research: the firefly *Photinus pyralis* luciferase, the sea pansy *Renilla reniformis* luciferase, and the marine copepod *Gaussia princeps* luciferase. Of these, the firefly luciferase is by far the most commonly utilized bioluminescent reporter. Firefly luciferase is 61 kDa in size and emits light at a wavelength of 562 nm (yellow to green), which is less-readily absorbed by tissues than the blue light (wavelength 480 nm) emitted by the smaller *Renilla* (36 kDa) and *Gaussia* (19.9 kDa) luciferases. Firefly and *Renilla* luciferases also remain intracellular, whereas *Gaussia* luciferase is secreted, making it more difficult to localize intracellular processes. The substrate of firefly luciferase, D-luciferin, also has a good pharmacokinetic profile and sufficient bioavailability. In mice, D-luciferin can be injected intraperitoneally and achieve a peak concentration in 10 min that remains stable for 30 min. Intranasal administration of D-luciferin also confers an increase in bioluminescence of one to two orders of magnitude in the nose and lungs of mice [43,44]. In contrast, the substrate of *Renilla* and *Gaussia* luciferases—coelenterazine—is not soluble in aqueous solutions and commonly precipitates when diluted in 100% alcohol. In animals, coelenterazine must be administered intravenously; it rapidly degrades in vivo, and it lacks the biodistribution profile of D-luciferin. 

More recently, a smaller luciferase NanoLuc (19.1 kDa) was engineered based on the luciferase from the deep-sea shrimp *Oplophorus gracilirostris* [15]. This enzyme catalyzes its substrate furimazine to generate high-intensity bioluminescence with greater than two-hour half-life and 150-fold higher specificity than the firefly and *Renilla* luciferases [15]. NanoLuc also exhibits improved physical stability and prolonged signal duration in vitro compared to firefly and *Renilla* luciferases. Because of its increased specificity and unique physical properties, NanoLuc bioluminescent reporter viruses hold promise for many in vivo imaging applications. However, like other marine luciferases, NanoLuc emits blue-shifted light (460 nm), which is more readily absorbed by tissues than the yellow-green light emitted by firefly luciferase. The NanoLuc substrate furimazine must also be administered intravenously, and its pharmacokinetic profile and toxicities have not been fully elucidated. For these reasons, the firefly luciferase is still often considered the preferred luciferase for live imaging applications.

### 4.8. Expression Strategies for Fluorescent and Bioluminescent Labels

**Figure 2:**
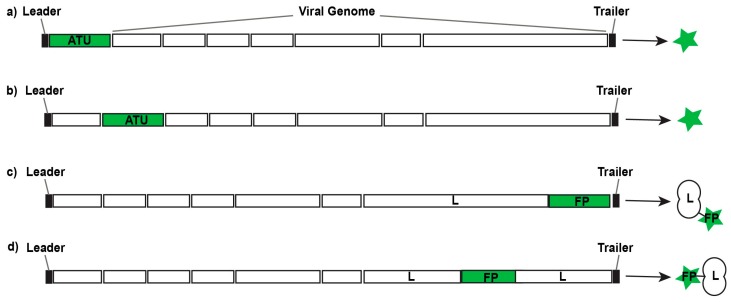
Design strategies for replication-competent fluorescent or bioluminescent reporter viruses. Additional transcription units (ATUs) can be inserted 3’ of the first gene in the viral genome (**a**) or 5’ of the final gene in the viral genome; between viral genes (**b**); or their sequences can be fused with viral genes to generate fusion proteins (FPs) either at the N-terminus or C-terminus (**c**) of a viral gene; or at inert hinge regions within a viral protein (**d**).

An additional consideration in the design of reporter viruses is the location of the reporter sequence within the viral genome. In general, a reporter protein can be encoded as an additional transcriptional unit (ATU) or as fusion protein with a viral gene product (Figure 2). Because paramyxoviruses are transcribed down a gradient according to proximity to the viral promoter, insertion of ATUs can decrease the expression of downstream genes and result in virus attenuation [45]. One strategy to address this has been to replace endogenous transcription initiation (gene start) signals downstream of the ATU with gene start signals known to be more efficient, to offset the inevitable disassociation of RNPs at gene junctions [46,47,48]. In paramyxoviruses, ATUs are most commonly placed upstream of the first gene position to preserve the transcriptional balance. Several groups have found that insertion of ATUs at this site was relatively well tolerated and resulted in little to no viral attenuation [49,50]. However, ATUs have been inserted into several other locations within the paramyxovirus genome, including between genes N and P [51]; P and M [44,52,53,54,55]; M and F [52]; and H and L [56,57], which have resulted in varying levels of attenuation. Van Remmerden et al. noted that insertion of an ATU encoding eGFP in the first gene position was attenuating, whereas insertion between the SH and G genes was not [58]. Unfortunately, few other studies have directly compared different expression strategies for fluorescent and bioluminescent reporter proteins, and many studies do not report attenuation levels in vitro or in vivo. 

Regarding influenza, the gene segments are relatively small (890 to 2341 nucleotides), which can make it difficult for them to accommodate large reporter gene sequences. One site which has been demonstrated to tolerate insertion of reporters is the C-terminus of the PB2 segment, where reporters have been incorporated as either PB2-fusion proteins [59] or as ATUs [60]. Other tolerant sites have included the C-termini of segments PB1 [61], NA [62], and PA [17]. Rearrangements of the influenza genome—e.g., to move NS2 downstream of PB1—also permitted insertion of GFP or *Gaussia* luciferase in the NS gene segment [63]. Reporters have also commonly been incorporated as fusion proteins with NS1 after Manicassamy et al. demonstrated this strategy in 2010 [64].

Fluorescent fusion proteins are commonly designed to study the actions of specific viral proteins. To minimize the steric hindrance of the reporter on the native protein, the fusion protein sequence is often inserted at the COOH- or NH-terminus of the gene [17,64], or within hinge regions [65] which may be biologically inert. Genetic linkers can then be added between the sequences [64] to further diminish steric hindrance. However, the reporter gene may have various effects on protein functions and interactions, which must be considered in the interpretation of results. One strategy recently employed to counteract the attenuating effects of reporter genes was the serial passage of reporter viruses in mice [66]. This strategy generated fluorescent viruses that grew to wild-type titers in mice and broadened the repertoire of fluorescent replication-competent reporter viruses for in vivo use.

## 5. Applications of Reporter Paramyxoviruses and Orthomyxoviruses

### 5.1. In Vitro Applications 

Fluorescent and bioluminescent reporter viruses have been developed for a variety of uses, ranging from basic science applications to anti-viral screening tools. The first fluorescent reporter paramyxovirus was a GFP-expressing measles virus used to track in vitro MeV spread in human astrocytoma cells [46]. Since then, fluorescent reporter paramyxoviruses have elucidated many aspects of viral pathogenesis, ranging from functional analysis of viral glycoproteins [67], to receptors for viral entry [49], the role of matrix protein [47], the trafficking of viral RNP [57], the role of surface glycoproteins on infection [68], and cellular tropism [69]. Influenza reporter viruses have likewise been utilized to elucidate various aspects of influenza pathogenesis [70,71]. Replacement of a non-essential protein with a fluorescent protein has been one strategy to study its effects on viral pathogenesis. Alternatively, the design of fusion proteins has enabled monitoring of specific viral protein localization and interaction [57,65]. Time-lapse confocal microscopy has also enabled the real-time visualization of intracellular processes with the help of fluorescent labels. 

As research tools, fluorescent and bioluminescent reporter viruses have also eased the process of viral detection and quantification. Titration of fluorescent viral foci or quantification of luminescence is often faster than standard plaque assays, which require several days for plaque formation. The correlation between titers obtained by standard methods and fluorescent or bioluminescent methods have been demonstrated in several models [16]. This characteristic has been exploited to generate new virus-neutralization assays which depend on the reduction of fluorescent foci rather than plaque or tissue culture infectious dose reduction [72]. Neutralization assays have also been developed using bioluminescent reporter proteins, which can be automated to measure the reduction of virally-expressed luciferase [73]. Such assays have been developed for influenza viruses [61,74], in addition to several paramyxoviruses including measles virus [75], mumps virus [55], HMPV [54], and RSV [72,73].

Another application of fluorescent reporter viruses as research tools has been in the generation of high-throughput assays to screen antiviral drugs and to measure susceptibility or acquisition of resistance. Such assays have been developed to screen for antivirals against single viruses [76,77] or multiple distinct viruses to identify broad-spectrum inhibitors. In 2013, Yan and colleagues developed a high-throughput assay to screen a library of antiviral compounds against multiple myxoviruses using firefly and *Renilla* luciferase reporter constructs. Their strategy identifiedbroad-spectrum anti-myxovirus compounds in addition to several paramyxovirus- and orthomyxovirus-specific hits [78]. They built upon this technology in 2015, when they synchronized influenza and RSV luciferase reporter expression kinetics to generate a more robust, high-throughput screen [79]. Such assays hold promise to accelerate the discovery of novel antiviral therapies.

### 5.2. In Vivo Applications 

Even in the early 2000s, fluorescently-labeled reporter paramxyoviruses were utilized for in vivo imaging of infection in small animal models. Using an eGFP-expressing measles virus, Duprex et al. imaged measles virus infection of the murine central nervous system [80]. This application has been extended to elucidate MeV target cells and tropism in vivo [56], particularly of oncolytic MeV [81], and to evaluate strategies to target and de-target oncolytic viruses for purposes of gene therapy [82]. Other paramyxoviruses that have been imaged in vivo using fluorescent reporters include Hendra virus [53] and RSV [44]. For RSV, one important application of this technology is to evaluate infectivity and attenuation levels of vaccine strains in animal models [72]. 

The first fluorescent influenza reporter virus was developed by Manicassamy et al. in 2010, who inserted a GFP reporter in the NS segment to generate an NS1-GFP fusion protein separated by a Gly-Ser-Gly-Gly (GSGG) linker [64]. This group tracked the dynamics of influenza virus infection in mice, identified target cells within the immune system, and measured the effects of antiviral compounds on in vivo infection dynamics. However, the GFP reporter virus did lack genetic stability and was attenuated in cell culture and in mice. To address these obstacles, Fukuyama et al. used a similar expression strategy to generate a reporter influenza expressing Venus, but subsequently serially passaged the virus in mice and identified deattenuated variants that had high genetic stability [66]. This group then generated a panel of mouse-adapted reporter viruses expressing one of four FPs: Venous, eGFP, eCFP, and mCherry. They called this panel of viruses “Color-Flu,” which they demonstrated could be co-imaged and utilized to track viral co-infections in animals. However, the application of these viruses to live, real-time imaging is limited by high background autofluorescence and attenuation of signal in vivo. 

Bioluminescent reporter viruses are advantageous in that they generate bright signals that can be detected by relatively inexpensive imaging technology. Because of the high signal-to-noise ratio, viruses can be imaged in live animals in real time. The first bioluminescent reporter virus to be utilized for in vivo imaging was herpes simplex virus 1 in 2002. Using firefly luciferase-labeled HSV-1, Luker et al. imaged viral spread in living mice and assessed response to antiviral therapy [13]. Among the paramyxoviruses, this technology has subsequently been applied to the live imaging of Sendai virus (murine parainfluenza type 1) [48] and RSV [44] in mouse models. Burke et al. engineered a firefly luciferase-labeled Sendai virus to image the spatial and temporal progression of infection and transmission following intranasal inoculation of mice. They found that transmissibility to naïve mice was associated with high viral replication in the upper respiratory tract and trachea of donors, but was independent of replication in the donor lower respiratory tracts. This study revealed a previously unknown dichotomy between parainfluenza virus transmissibility and pathogenesis in the mouse model. Bioluminescent imaging has similarly been used to image influenza virus infection dynamics and transmission in mice [17,60,62]. In 2015, Karlsson and colleagues utilized bioluminescence to image a NanoLuc-labeled influenza virus in ferrets, demonstrating the potential of this approach to assess tissue distribution and transmissibility of infection in larger animal models [16].

## 6. Future Directions

Fluorescent and bioluminescent reporter viruses have revolutionized our understanding of molecular virology and aspects of viral tropism and transmission. These reporters have also enabled the development of high-throughput assays, which have greatly reduced the time and effort necessary to quantify viral infection, to screen antiviral compounds and antibodies, and to perform neutralization assays. Although there is still no one-size-fits-all approach to the design of reporter viruses, new fluorescent and bioluminescent labels continue to be developed which have more optimal characteristics for labeling and detection. The development of new far-red and near infrared fluorescent proteins holds promise for the future of live fluorescent imaging. In addition, improvements in bioluminescent imaging technology could help hone spatial resolution of this already powerful modality.
